# *Nigella sativa* Oil Enhances the Spatial Working Memory Performance of Rats on a Radial Arm Maze

**DOI:** 10.1155/2013/180598

**Published:** 2013-12-26

**Authors:** Mohamad Khairul Azali Sahak, Abdul Majid Mohamed, Noor Hashida Hashim, Durriyyah Sharifah Hasan Adli

**Affiliations:** ^1^Division of Biohealth Science, Institute of Biological Sciences, Faculty of Science, University of Malaya, 50603 Kuala Lumpur, Malaysia; ^2^Division of Biology, Center for Foundation Studies in Science, University of Malaya, 50603 Kuala Lumpur, Malaysia

## Abstract

*Nigella sativa*, an established historical and religion-based remedy for a wide range of health problems, is a herbal medicine known to have antioxidant and neuroprotective effects. This present study investigated the effect of *Nigella sativa* oil (NSO) administration on the spatial memory performance (SMP) of male adult rats using eight-arm radial arm maze (RAM). Twelve *Sprague Dawley* rats (7–9 weeks old) were force-fed daily with 6.0 **μ**L/100 g body weight of *Nigella sativa* oil (NSO group; *n* = 6) or 0.1 mL/100 g body weight of corn oil (control) (CO group; *n* = 6) for a period of 20 consecutive weeks. For each weekly evaluation of SMP, one day food-deprived rats were tested by allowing each of them 3 minutes to explore the RAM for food as their rewards. Similar to the control group, the SMP of the treated group was not hindered, as indicated by the establishment of the reference and working memory components of the spatial memory. The results demonstrated that lesser mean numbers of error were observed for the NSO-treated group in both parameters as compared to the CO-treated group. NSO could therefore enhance the learning and memory abilities of the rats; there was a significant decrease in the overall mean number of working memory error (WME) in the NSO-treated group.

## 1. Introduction

*Nigella sativa* is a dicotyledonous medicinal plant, belonging to the family Ranunculaceae, which is native to southern Europe, North Africa, and Asia Minor, and is widely cultivated in Pakistan and India. The plant reproduces asexually and forms capsulated fruits containing numerous white trigonal seeds, when being exposed to air it turns black thus commonly known as Black Seed [1, 2]. Among the Muslim and Arabian communities, it is well known as *Habbat Albarakah*, *Alhabahat Alsawda*, and *Alkamoun Alaswad*. It is also referred to by various names in different languages, for instance, *Shuniz*, *Khodhira*, Black Cumin, or even as Black Caraway [3].

As an established historical and religion-based remedy for a wide range of health problems, it is one of the herbal medicines that is being actively investigated and is thus gaining worldwide recognition [4]. Reviews have reported *Nigella sativa* as having antioxidant and neuroprotective effects in addition to many other therapeutic effects, such as antitumor, immunopotentiation, anti-inflammatory, and antimicrobial [2, 5, 6]. Consuming antioxidant nutrients, such as* Nigella sativa*, could be one of the promising health strategies to help prevent the oxidative damage to cells, particularly in the brain regions which are related to memory functions.

The radial arm maze (RAM) is one of the standard apparatuses used in behavioural-based research, commonly using rats as experimental animals to assess spatial memory. Spatial memory, either its working memory and/or reference memory components, refers to memory for spatial information by which the brain works in recognizing, codifying, storing, and recovering information about objects or routes. The spatial memory recognitions have always been related to exploratory behaviour and curiosity. This kind of behaviour may represent the need to acquire information when subjects face new environments [7]. Previous studies on the effects of *Nigella sativa* seeds and its constituents, on the central nervous system, and on behavioural actions are limited, as compared to similar studies using other plant materials [8]. In view of this, the present study was designed to investigate the possible beneficial effects of *Nigella sativa* oil (NSO) administration on the spatial memory performance (SMP) of male adult rats, using the RAM apparatus.

## 2. Methods

### 2.1. Experimental Animals, Housing, and Mode of Treatment

Twelve male adult *Sprague Dawley* rats aged 7–9 weeks old (weighing from 200–240 g) were acquired from the University of Malaya Laboratory Animal Centre and allowed to acclimatize to 12 hours of light and 12 hours of darkness per day. Individually housed rats in polypropylene cages, with steel wire tops and regularly changed layers of sawdust at the bases to absorb urine, were provided with sufficient standard chow pellets (20–30 g) daily and water *ad libitum*. The rats were randomly assigned to two groups of treatments, each being force-fed with (a) *Nigella sativa* oil (6.0 *μ*L/100 g body weight) (NSO group; *n* = 6) and (b) corn oil (control) (0.1 mL/100 g body weight) (CO group; *n* = 6). They were treated for five days a week for a period of 20 consecutive weeks (100 days). Phases 1–4 (weeks 1–8; with 2 weeks per phase) were considered as the acute treatment period, and phases 5–10 (weeks 9–20) were considered as the chronic treatment period. On day 6 of each week, the rats were deprived of food although water was continuously provided. RAM test was conducted on day 7 of the week. All procedures were carried out in compliance with the guidelines as stipulated by the Institutional Animal Care and Use Committee (IACUC), University of Malaya [ISB/20/04/2012/DSHA (R)].

### 2.2. Apparatus

Each behavioural testing session was conducted in a standard RAM consisting of a central platform 25 cm in diameter with eight arms of 70 cm (length) × 10 cm (width) × 15 cm (height) each, radiating equiangular from the central platform that served as a starting base. The white-painted maze was placed at a fixed position to reduce the variability of each test.

### 2.3. RAM Testing

In the present study, baited and unbaited arms were fixed throughout the tests. The 1st, 3rd, 6th, and 8th arms were baited while the 2nd, 4th, 5th, and 7th arms were unbaited. At the very beginning of each test session, each rat was placed in the central starting platform of the RAM in the position facing towards the 1st arm. Food-deprived rats were expected to seek specific arms with rewards and subsequently register and retain the memory of each entered arm where food was present. Each rat was allowed to freely explore and consume food rewards for 3 minutes or until all food rewards of the four baited arms were eaten, whichever occurred first. An entry was recorded every time the rat placed all four paws into the initial part of the arm. The maze was then thoroughly cleaned with 70% alcohol prior to the next test session in order to minimize the effect of residual odours from the previous test.

### 2.4. Scoring of Behaviour

The first entry into never-baited arms was scored as a reference memory error (RME) and reentry into arms where the food reward had already been eaten was scored as a working memory error (WME) [9].

### 2.5. Statistical Analyses

The data gathered were subjected to Analyses of Variances (ANOVA). The analyses were performed using the SPSS statistical software for Windows Version 20.0 (SPSS Inc., Chicago, IL, 2011). The least squares means and standard errors were then used to plot the curves for the NSO and CO groups. *P* < 0.05 was regarded as statistically significant.

## 3. Results

**Figure 1:**
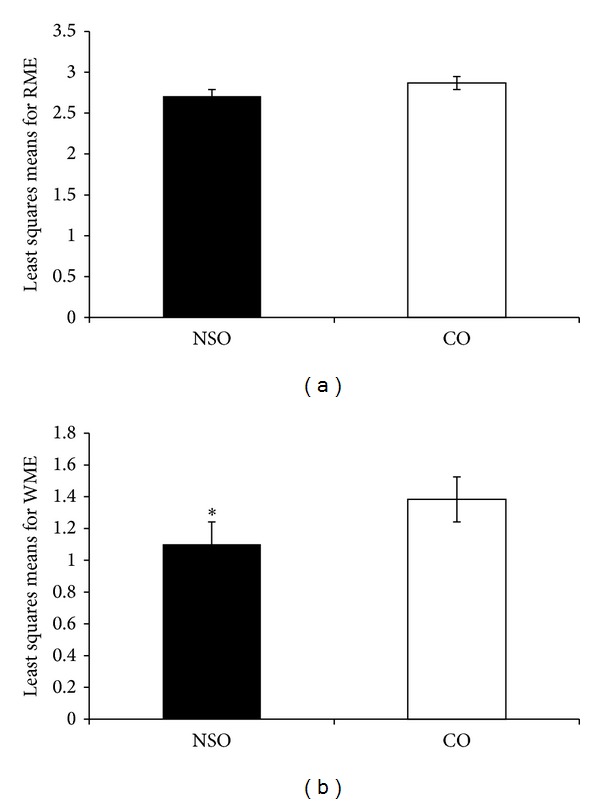
Least squares means and significance level of the effects of NSO and CO (control) on RAM spatial memory performance (SMP): (a) reference memory error (RME) and (b) working memory error (WME). Asterisk denotes significant difference from control (*P* < 0.05).

**Figure 2:**
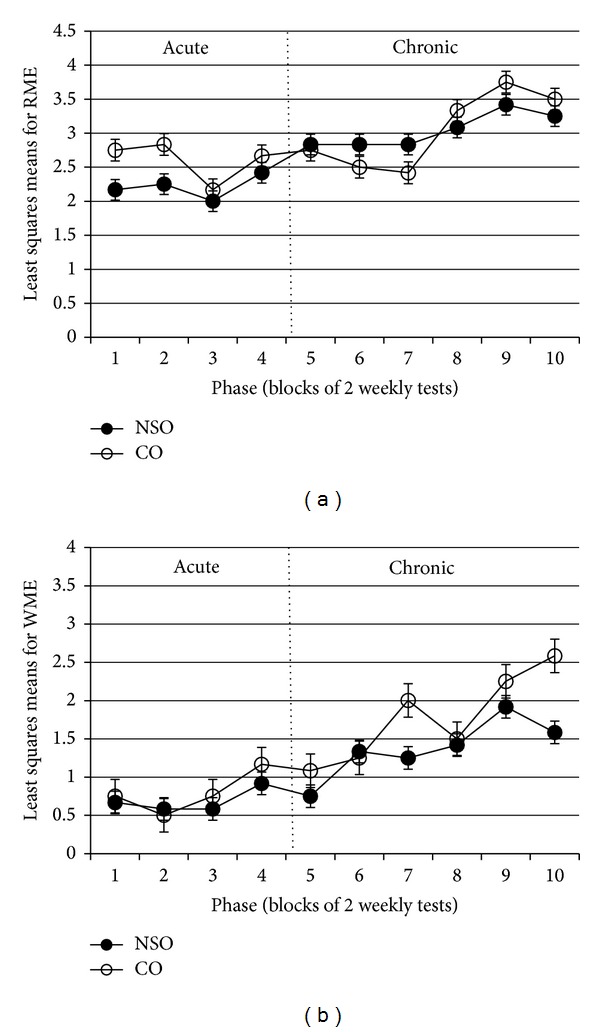
Least squares means and standard errors for (a) reference memory error (RME) and (b) working memory error (WME) of spatial memory performance (SMP) of rats treated with NSO and CO for 10 phases.

Mean squares from ANOVA of RME and WME of SMP for the effects of treatment, phase, and the interaction between treatment and phase are shown in Table 1. The interactions between treatment and phase were not significant (*P* > 0.05) for both parameters (Table 1). Treatment effect was not significant (*P* > 0.05) for RME but was significant (*P* < 0.05) for WME, specifically involving NSO (Figure 1(b)). Phase had a significant effect (*P* < 0.05) on both parameters, indicating that the phase of treatment influenced the number of errors committed during the experiment. The errors in the acute period are less than in the chronic period as shown in Figures 2(a) and 2(b). The least squares means from the ANOVA for the interaction effects were used to plot the curves shown in these figures.

For both RME (Figure 2(a)) and WME (Figure 2(b)), the CO-treated rats generally showed more errors. In addition, least squares means for WME were lower than RME for both NSO- and CO-treated groups. Figure 2(a) showed that, in general, RME decreased within the first three weeks and increased from then onwards. No significant difference was detected between the NSO- and CO-treated rats. Although there were cases of crossing of the interaction effects in phases 6 and 7 for the RME and WME parameters, it should be reiterated that the interaction effects were not significant for both parameters.

## 4. Discussion

Generally, SMP parameters of RME and WME of the NSO-treated group were not hindered as there were lesser mean numbers of error throughout the experiment compared to the CO-treated group. It is also reasonable to suggest that treatment with NSO could enhance the learning ability and memory of the rats. The present study demonstrated that there was a significant decrease in the overall mean number of WME in the NSO-treated group, but not for the RME parameter. Working memory is also normally referred to as “short-term memory” as it allows temporal storage of a limited amount of spatial information and keeps it available for immediate access. SMP throughout the 10 phases of the experiment showed no significant difference between each phase for both RME and WME in the NSO-treated group as compared to the CO-treated group.

Worth nothing is that in this study there was a declining trend in both RME and WME in the acute treatment period. This was possibly due to the result of learning and facilitatory effects of NSO. In addition to that finding, RME was possibly reduced because foraging behaviour, a common behaviour reflecting anxiety when confronted with novelty in unfamiliar spatial settings, was no longer a motivating factor in an environment that was getting to be progressively familiar to the rats [10].

However, absence of overall declining trend of errors after NSO chronic administration might possibly be due to the duration of treatment. Through 100 days of treatment, the rats might have developed other strategies to solve the maze, such as memory of the response strategy and direction of turning rather than using their spatial memory system [11]. In this case, the rats would only enter the adjacent arms without considering whether it was baited or unbaited, thus affecting the results. It has been hypothesized that captivity could reduce the rats' environmental complexity, thereby, restricting their physical movements and spatial memory-based experiences [12]. Additionally, the rats were likely to experience stressful conditions due to the long captivity period involved in chronic treatment. Stress is known to disrupt good memory formation through retroactive interference effects, including housing and environmental disturbances [13]. Although there are studies which have postulated that spatial memory in rats appears resistant to disruption when the interpolated (“between-tests”) event is different from the original learned task, interference still can occur if there is a large amount of interpolated experience, or if the interpolated event is very similar to the original task [14]. Another possibility of the results seen in the chronic phase, especially in relation to RME, would be because the rats were attentively exploring the RAM to seek rewards (food). However, if negative reinforcement had been used, the results could be different than what was obtained. Thus, the facilitating effect of NSO on memory, as best demonstrated for WME, was countered by the above-mentioned factors.

The beneficial effects of *Nigella sativa* on memory are most likely due to the ability of one or more of its constituents to protect against cellular damage caused by oxidative stress through its free radical scavenging properties. Notably, acetylcholine (ACh) as a neurotransmitter plays a role in facilitating learning and memory, and therefore, its decreased release will result in memory impairment [15]. Pharmacological studies demonstrated that *Nigella sativa* could be involved in acetylcholinesterase (AChE) activity inhibition, thus retaining the effects of ACh in the encoding of new memories.

A previous study demonstrated that NSO chronic oral administration could enhance the consolidation and recall capability of stored information and spatial memory in diabetic animals [16]. The effects were indicated through the use of passive avoidance and Y-maze tests, improved initial latency, step-through latency, and alternation behaviour. In addition, a study has also reported that extract of *Nigella sativa* could prevent scopolamine-induced deficit memory in rats, as the animals showed better performance in passive avoidance tests and decreased AChE activity in the hippocampus and cortex tissue of the brain [17]. A recent study by El-Marasy and his colleagues [18] reported that oral pre-treatment of NSO significantly reversed the amnesic effect of scopolamine-induced deficit of spatial and nonspatial working memory impairment in the T-maze alternation task and object recognition test, respectively. Interestingly, NSO tended to mimic the effects of donepezil, an AChE inhibitor, which is known to have positive effects by decreasing malondialdehyde (MDA) and brain tumor necrosis factor-alpha (TNF-*α*) content as well as increasing glutathione brain contents. These findings, therefore, point to the possible neuroprotective properties of NSO through its antioxidant activity.

## 5. Conclusion

The current study suggests that in both the acute and chronic administrations, NSO could enhance working memory (short-term memory) in the RAM performance of rats. In addition, it was found that NSO facilitated SMP in RAM tests, particularly in the acute treatment phase (40 days), which was indicated by the declining trend of the mean numbers of error.

## Figures and Tables

**Table 1:** Mean squares from Analyses of Variances (ANOVA) for reference memory error (RME) and working memory error (WME) on RAM spatial memory performance (SMP) of rats treated with *Nigella sativa* oil (NSO) and corn oil (CO) for 10 phases.

		Mean squares	
		RME	WME
Treatment	1	1.504	4.817*
Phase	9	5.125*	7.637*
Treatment × phase	9	0.699	0.733
Error	220	1.238	0.981

Asterisks denote significant difference (*P* < 0.05).
